# Fauna Antiqua Sivalensis; Being the Fossil Zoology of the Sewalik Hills, in the North of India

**Published:** 1846-04

**Authors:** 


### ART. XIII.

*Fauna Antiqua Sivalensis; being the Fossil Zoology of the Sewalik Hills, in the North of India.* By HUGH FALCONER, M.D., F.R.S., F.L.S., F.G.S., &c. &c., and PROBY T. CAUTLEY, F.G.S., Captain in the Bengal Artillery, &c. &c. Part I.—*London,* 1845. 8vo. pp. 64. With Twelve Lithographic Plates, large folio.

ALTHOUGH this is but the first number of a work which will not be completed in less than twelve, and which may very probably extend to more, we think it right to bring it thus early under the notice of our readers; both in order that we may do what in us lies to make it extensively known, and that we may enjoy the pleasure of paying our humble tribute of praise to its extraordinary merits, or rather to the merit of those researches of which it is one of the results or exponents,—a still more brilliant result and a yet more satisfactory exponent being the magnificent collection of fossil remains deposited in the British Museum, and now in course of arrangement under the superintendence of Dr. Falconer.

"The object of this publication," to use the words of its authors in their prospectus, "is to make known, in a connected and complete series, the numerous fossil animals which have been discovered in the North of India, by the authors and other inquirers, during the last twelve years; and to develop the bearings of these discoveries on the physical and geological history of India, during a great part of the tertiary period. The fossil fauna of the Sewalik range of hills skirting the southern base of the Himalayahs has proved more abundant in genera and species than that of any other region yet explored. As a general expression of the leading features, it may be stated, that it appears to have been composed of representative forms of all ages, from the oldest of the tertiary period down to the modern; and of all the geographical divisons of the Old Continent, grouped together into one comprehensive fauna in India. Of the forms contained in it may be enumerated, in the *pachydermata,* several species of mastodon and elephant, the hexaprotodon hippopotami, merycopotamus, rhinoceros, anoplotherium, sus, and three species of equus; in the *ruminantia,* the colossal genus sivatherium, peculiar to India, with species of camelus, camelopardalis, bos, cervus, and antelope; in the *carnivora,* species of most of the great types, together with several remarkable undescribed genera; in the *rodentia,* several species; in the *quadrumana,* several species; in the *reptilia,* the gigantic tortoise (colossochelys), with species of emys and trionyx, and several forms of crocodile. To these may be added the fossil remains of birds, fishes, crustacea, and mollusca."

Of all these remains those of the colossochelys are perhaps the most *wonderful,* since the idea of a tortoise twenty-two feet long (over the arch of the carapace), from the snout to the tail, is more completely foreign to our ordinary notions regarding that group of animals, than would be the description of an elephant standing thirty feet high, or of a hippopotamus measuring as much round the belly,—accustomed as we are to connect the very names of these animals with the idea of gigantic size. But the most *interesting* of these remains, to the zoologist and comparative anatomist, are such as tend to fill up the hiatuses left in various parts of the existing series, to indicate affinities where no near relationship previously seemed to exist, and to show how every conceivable union of organs, that could possibly coexist, finds its realization in nature. And to the geologist, the mode in which these various remains have been imbedded,—the cause of the wonderful aggregation, not merely of individuals but of species, and not merely of species that may be presumed to have co-existed, but of many that must have succeeded one another at very remote periods of time,—and the past condition of the part of the Eastern Continent in which they present themselves,—are problems of the highest interest, towards the elucidation of which we have reason to believe that the authors will be enabled to contribute a vast amount of novel and important information, from the varied stores which they have collected during their laborious and protracted inquiries.

The authors further tell us, that "they have been induced to undertake the work by the belief that the scientific reputation of this country and the credit of the Indian service are concerned in bringing to light researches embracing so many new facts, and bearing so importantly on the past physical history of the vast possessions of the British Empire in India. They are not insensible to the difficulty and extent of the subject, but they hope that they are in some measure prepared for it, by previous investigations extending through several years."

In this truly modest spirit do they allude to a series of researches, carried on, we have reason to know, under almost every possible disadvantage, with the most indomitable perseverance. At a distance of many hundreds of miles from those sources of information which the metropolis of Anglo-India might afford, with no copy of the 'Ossemens Fossiles' to guide their identifications, by the light which Cuvier had struck out, with no Museum, such as that of the Jardin des Plantes, or of our own College, to which to have continual recourse for the comprehension of the nature of their fast-accumulating treasures, they were thrown upon the book of nature, and upon the living museums of the neighbouring woods and plains, for the determination of these fossils, by the aid of their existing congeners; and their own guns, whilst providing daily food for their corporeal wants, supplied also their intellectual provender,—namely, the dry bones, which to all else, save the fierce hyæna, were valueless, but which to them were in place of books and plates and professors skilled in the lore of comparative anatomy. We rejoice to learn that the British Government and the Directors of the East India Company have thought fit to accord such an amount of aid *in limine*, as will ensure the successful progress of this truly national undertaking; and we also rejoice in the hope that the two hundred chests of Sewalik fossils, which have been presented by Capt. Cautley to the British Museum, will be speedily arranged there by Dr. Falconer, and made fully accessible to the public.

The style in which the work is produced, and the exceeding beauty of the illustrations, do great credit to the enterprising publisher, and to the artists who have been employed upon them. The lithographs will bear the closest inspection, and challenge comparison with any that have proceeded from continental presses, distinguished as the latter have always been for correctness of drawing and beauty of finish.

Following the order of the 'Ossemens Fossiles,' the group of proboscidea is first brought under review; and its selection is the more appropriate, as it most signally displays the numerical richness of forms, which characterizes the fossil fauna of India; whilst the materials collected by the authors are most valuable, as tending to settle many points which have hitherto been subjects of grave discussion. Palæontology made its first great advance in the exact determination of the mammoth of Siberia, and of the mastodon of North America, which we owe to Cuvier, and the new forms which have been discovered since that time, have been the subjects of attentive examination by Owen, Blainville, and other eminent anatomists.

"But notwithstanding the vast amount of observation on the subject during late years, a great difference of opinion has prevailed among comparative anatomists and palæontologists, down even to the period when we now write, in regard to the degree of affinity and the generic relations of the different species of mastodon and elephant. The majority of late authorities, including Cuvier and Owen, have regarded them as constituting two distinct and well-marked, though closely-allied, genera; others have gone the length of breaking up mastodon into two genera: while M. de Blainville has reverted to the opinion of some of the earlier observers, that the so-called mastodons and elephants are but modifications of one common type, differing so little from each other that all the species may, with propriety, be included within the limits of a single genus. A still greater and vastly more important difference of opinion has prevailed, regarding the number and characters of the species; for, while the conflicting views respecting the generic distinctions concern little more than the principles of systematic classification, the accurate determination of the fossil species affects the value of facts, which implicate the accuracy of some of the most weighty arguments in the geology of the later tertiary strata, more especially such as relate to the changes of climate which are supposed to have accompanied their deposition, and the extension of the species through a wide range of time and space." (p. 3.)

Thus Cuvier regarded all the fossil-elephant remains of the Old and New World as belonging to one and the same species, the *elephas primigenius* or mammoth, and Professor Owen has been of the same opinion. By M. de Blainville it has even been urged that the mammoth cannot be shown to be specifically distinct from the existing Indian elephant. Other palæontologists, on the contrary, have constructed no fewer than ten species out of the single species of Cuvier; founding the distinctive characters upon the differences presented by the molar teeth. So in regard to the mastodons, some would restrict the number of species (known previously to those now described) to four or five, whilst others would raise the number to twenty. This great diversity of opinion, almost unequalled in any other section of mammalian palæontology, has, in a great measure, arisen from the isolated and defective nature of the materials relating to this tribe, as they ordinarily come before the palæontologist, and especially from the peculiarities which characterize the dentition of the proboscideans, and which cause the teeth of the same species to present very different aspects at different periods. It is only, therefore, as our authors justly observe, from the comparison of an extensive series of specimens, embracing every period of life, and the range of individual sexual varieties through which the species runs, that any safe conclusions can be drawn regarding the distinctive characters of any one form. In this respect they have been peculiarly fortunate, owing to the surprising number of forms belonging to this family embraced in the fossil fauna of India, and the immense abundance in which their remains have been met with. The results of their researches lead them to differ on certain points from all palæontologists who have treated of this family. As the subject is only commenced, however, in the present Part, we shall not now enter upon it, but shall present our readers with a general summary of our author's views, and of the grounds of them, when they shall have been completely developed.

---

